# Supplementary material for: The grape powdery mildew resistance loci Ren2, Ren3, Ren4D, Ren4U, Run1, Run1.2b, Run2.1, and Run2.2 activate different transcriptional responses to Erysiphe necator
Source: Front Plant Sci. 2022 Dec 19;13:1096862. doi: 10.3389/fpls.2022.1096862 (PMC9806207; doi:10.3389/fpls.2022.1096862)
Supplement: Supplementary file 9 [file DataSheet_2.docx]

**Supplementary Figure 1**: (**A**) Visual scores of powdery mildew infection rate in each grape accession. Disease development was rated using a 5-point scale, 0 (no symptom) to 4 (fungal structures cover majority of the leaves). Significant differences detected with Kruskal-Wallis test followed by post hoc Dunn's test (*P* ≤ 0.05) are indicated by different letters. (**B**) Correlation between *E. necator* log_2_-TPM per sample and visual scores of disease development. Linear trend (dark red line), 95% confidence interval (gray shading), 95% prediction interval (dark blue dotted line), squared correlation coefficient factor (R^2^) and *P* value (*P*) are provided.

**Supplementary Figure 2**: Matrix displaying kinship coefficients between the fourteen genotypes based on SNPs and INDELs relative to PN40024. At the top, we show the hierarchical clustering based on kinship coefficients.

**Supplementary Figure 3**: (**A**) Number of *de novo* assembled grape CDS per accession. (**B**) Cluster dendrogram based on the presence/absence of grape CDS. Dendrogram was generated by converting Pearson correlation coefficients of pairwise comparison of *de novo* assembled grape CDS into distance coefficients.


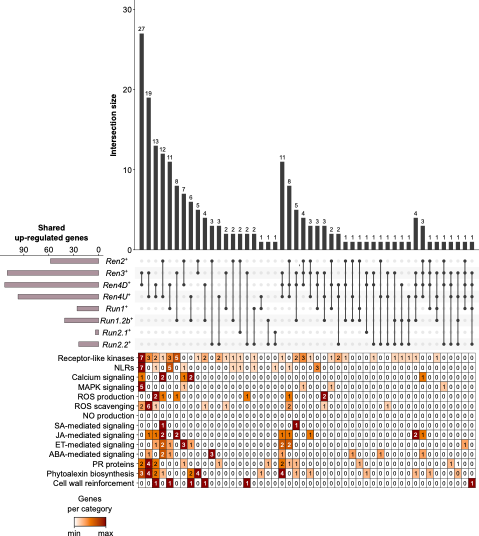


**Supplementary Figure 4**: Comparison of the defense-related genes found up-regulated in response to *E. necator* at 1 dpi only in PM-resistant accessions.

**Supplementary Figure 5**: Comparison of the defense-related genes found down-regulated in response to *E. necator* at 1 dpi only in PM-resistant accessions.

**Supplementary Figure 6**: Comparison of the defense-related genes found up-regulated in response to *E. necator* at 5 dpi only in PM-resistant accessions.

**Supplementary Figure 7**: Comparison of the defense-related genes found down-regulated in response to *E. necator* at 5 dpi only in PM-resistant accessions.
